# Improving Large Language Model Applications in the Medical and Nursing Domains With Retrieval-Augmented Generation: Scoping Review

**DOI:** 10.2196/80557

**Published:** 2025-10-21

**Authors:** Yiqun Miao, Yuhan Zhao, Yuan Luo, Huiying Wang, Ying Wu

**Affiliations:** 1 School of Nursing Capital Medical University Bejing China; 2 The Chinese Institutes for Medical Research Bejing China

**Keywords:** large language model, retrieval-augmented generation, medical domain, nursing, scoping review, artificial intelligence, AI

## Abstract

**Background:**

Retrieval-augmented generation (RAG) is increasingly used to improve large language models in the medical and nursing domains. However, a comprehensive understanding of its specific architecture and applications in medical and nursing reasoning remains limited.

**Objective:**

We aimed to summarize the current state, existing limitations, and future development directions of RAG in the medical and nursing domains.

**Methods:**

The PubMed, Web of Science, IEEE Xplore, and arXiv databases were searched for relevant articles using queries that combined terms related to RAG, medical, and nursing domains, covering the period from November 1, 2022, to May 31, 2025. This review was conducted following the PRISMA-ScR (Preferred Reporting Items for Systematic Reviews and Meta-Analyses Extension for Scoping Reviews) guidelines.

**Results:**

A total of 917 articles were retrieved, of which 67 met the inclusion criteria. Most studies focused on the medical domain (63/67, 94%), while only a few addressed nursing applications (4/67, 6%). The RAG frameworks included in this review were categorized into 5 functional types: text-based RAG (36/67, 54%), knowledge graph–enhanced RAG (17/67, 25%), agentic RAG (6/67, 9%), multimodal RAG (2/67, 3%), and plug-and-play RAG (6/67, 9%). On the basis of the Simon decision-making process theory, we divided the RAG workflow into 4 stages: intent recognition, knowledge retrieval, knowledge integration, and generation. Only 26 studies included explicit reasoning support, and few were aligned with real-world clinical workflows. Only 12 studies attempted to address ethical considerations related to RAG.

**Conclusions:**

We identified 4 key shifts in recent RAG development: shifting from surface-level matching toward contextualized intent recognition, from vague semantics toward logic-driven dynamic retrieval, from passive toward active knowledge retrieval, and from simple aggregation toward coherent context construction. However, most RAG systems in the medical and nursing domains have not yet introduced reasoning methods, and those that have are still predominantly reliant on data‑driven associations without causal modeling. This highlights the need to integrate causal mechanisms for more effective and domain-relevant reasoning in health care.

**Trial Registration:**

OSF Registries 10.17605/OSF.IO/WBSV5; https://osf.io/wbsv5

## Introduction

### Background

Large language models (LLMs) represent a breakthrough in artificial intelligence (AI), capable of processing, understanding, and generating humanlike language at scale. With their advanced natural language processing capabilities, LLMs are increasingly explored in specialized domains, including both the medical and nursing fields [1]. Recent studies have demonstrated the potential of LLMs to support a wide range of clinical tasks, such as diagnosis support, medical documentation, and treatment planning for medical professionals, while also showing promise in assisting nursing-specific duties, such as care plan generation, patient education, and automation of nursing notes [2,3].

Despite the potential of LLMs, their integration into clinical and nursing practice is hindered by several critical challenges. A key concern is the generation of inaccurate content, along with limited transparency regarding how responses are produced. However, whether in medical applications or nursing practice, even minor errors can have a serious impact on patient safety [4]. Furthermore, because LLMs do not inherently access external knowledge bases, their outputs may fail to incorporate the latest evidence. This includes clinical guidelines and drug updates that are critical for medical decision-making, as well as nursing best practices and care protocols that are essential for effective patient management [5]. To address the limitations of “out-of-the-box” LLMs, Lewis et al [6] proposed retrieval-augmented generation (RAG) for knowledge-intensive natural language processing tasks.

RAG enhances the generative capabilities of LLMs by incorporating external knowledge retrieval mechanisms [7]. Unlike traditional models relying solely on internal parameters, RAG leverages in-context learning to proactively retrieve relevant information before response generation [8]. This significantly reduces inaccurate information and improves the transparency of information sources, which is crucial in health care [9]. Furthermore, as medical and nursing scenarios involve distinct reasoning paradigms, general-purpose LLMs often struggle to differentiate between them. RAG addresses this limitation by supporting diagnosis-centered medical reasoning through context-aware retrieval of evidence-based knowledge and facilitating nursing reasoning through the integration of patient information to assist nurses in identifying cues and confirming nursing problems, thus providing differentiated support for both paradigms [10,11]. However, current reviews of RAG primarily adopt a technical perspective while overlooking the specific needs and contexts of medical and nursing practice, such as alignment with clinical workflows, adherence to ethical standards, and the ability to reason as clinicians or nurses [12,13].

### Goals of This Review

To bridge this knowledge gap and enable its effective and responsible integration, developing a comprehensive understanding of current applications of RAG in medical and nursing settings is crucial. Through this scoping review, we aim to categorize types of RAG and their developmental stages, while establishing a foundational understanding of the field in terms of adopted techniques, reasoning strategies, application tasks, and ethics. This review serves a dual purpose: first, to provide health care professionals with a navigational map of existing research and second, to identify key trends, limitations, and future directions of RAG in the medical and nursing domains. Considering the complexity and fragmented information landscape, where implementation is often driven by technical teams unfamiliar with clinical workflows, this study takes an important step toward enabling health care professionals to lead RAG system development and application.

## Methods

### Overview

This scoping review included articles that described the development or application of RAG technologies in medical and nursing contexts. The review followed the methodological framework proposed by Arksey and O’Malley [14] and subsequently refined by Levac et al [15]. This methodological framework consists of five stages: (1) identifying the research questions, (2) identifying relevant studies, (3) selecting studies, (4) charting the data, and (5) collating, summarizing, and reporting the results. The PRISMA-ScR (Preferred Reporting Items for Systematic Reviews and Meta-Analyses extension for Scoping Reviews) checklist [16] was used as a guideline in reporting the results of the study (Multimedia Appendix 1). This project was registered with the Open Science Framework [17].

### Identifying the Research Questions

To address the aims of the study, the following research questions were identified:

1. Into what categories can RAG frameworks in the medical and nursing domains be classified?
2. Can the workflow of RAG systems be structured into distinct stages to guide medical and nursing practice, and what enhancement techniques are applied at each stage?
3. What methods have been used to improve reasoning capabilities within RAG frameworks?
4. In what application tasks have medical and nursing RAG frameworks been deployed?
5. What practical measures have been taken to mitigate ethical risks in the development and application of RAG frameworks in the medical and nursing domains?

### Identifying Relevant Studies

We conducted a literature search using 4 electronic databases covering the period from November 1, 2022, to May 31, 2025: PubMed, Web of Science, IEEE Xplore, and arXiv. This time frame was chosen because LLMs only became widely available in late 2022, and RAG was introduced to reduce the generation of inaccurate content. In light of the rapid development in this field, preprints were also included to ensure the inclusion of the most recent advances. A comprehensive search strategy was developed and refined in collaboration with the research team, and a health science librarian was consulted. Search terms included keywords such as *retrieval augmented generation*, *RAG*, *health care*, *medicine*, *medical*, *nursing*, and *care*. The complete search strategy is provided in Multimedia Appendix 2.

### Study Selection

The initial criteria used to identify articles included (1) studies published in English only, to ensure consistency in data extraction and interpretation, as translating non-English studies could introduce potential biases or inaccuracies that might affect the overall findings; (2) studies applying the RAG framework to perform end-to-end or user-facing medical tasks were included while those focusing solely on isolated natural language processing components, such as entity recognition or relation extraction, were excluded because the aim was to explore the integrated technical architecture and application of complete RAG systems rather than individual submodules; (3) only studies proposing RAG frameworks applied to medical and nursing domains were considered; (4) studies were required to clearly describe the RAG framework architecture, the retrieval data sources, and the retrieval methods used. In addition, we excluded literature reviews, conference abstracts without accessible full text, and articles without accessible full text.

### Data Extraction

Identified articles were imported into EndNote (Clarivate Inc), where duplicates were removed. Titles and abstracts were screened independently and categorized as *include*, *exclude*, or *potentially include*. Two authors conducted independent assessments, and any disagreements were resolved through discussion and consensus, with a third reviewer adjudicating if a consensus could not be reached. A standardized data extraction form was developed and refined based on team feedback. We initially conducted a pilot extraction on 10 representative studies to explore and determine the most appropriate data extraction dimensions for this review. On the basis of the findings from this pilot phase, the extraction categories were developed a priori and further refined. Ultimately, the extracted data covered 5 key dimensions: the type of RAG method proposed, technical details corresponding to each stage of the RAG framework, reasoning strategies used, application tasks addressed, and ethical considerations reported. Relevant information was extracted for each included article, with one reviewer performing the initial extraction and another verifying and completing the data as needed.

### Quality Assessment

To evaluate the reporting quality of the included RAG-related studies, we adopted the Minimum Information for Medical AI Reporting (MINIMAR) framework [18], a recently developed guideline specifically tailored for the reporting of AI research in medical contexts. Although many of the included studies were proof of concept, the MINIMAR framework was ultimately selected for evaluation because it specifically addresses the critical aspects of medical AI systems, such as data transparency, model evaluation, and other related factors. MINIMAR outlines four essential components: (1) study population and setting, (2) patient demographics, (3) model architecture, and (4) model evaluation, comprising a total of 21 key reporting features correspond to all four components of the MINIMAR framework. The overall MINIMAR adherence rate was subsequently calculated to quantify the reporting quality across studies. Given the rapid development of RAG-related research, no standardized quality appraisal tool currently exists in this field. While MINIMAR assesses reporting completeness, it does not cover methodological rigor or clinical relevance. To address this gap, we additionally applied a self-developed evaluation framework, including 3 dimensions: methodological rigor, clinical relevance, and reporting transparency, with a total score of 10 points. All studies were independently assessed by 2 reviewers, with disagreements resolved through discussion.

## Results

### Overview of Included Studies

**Figure 1:**
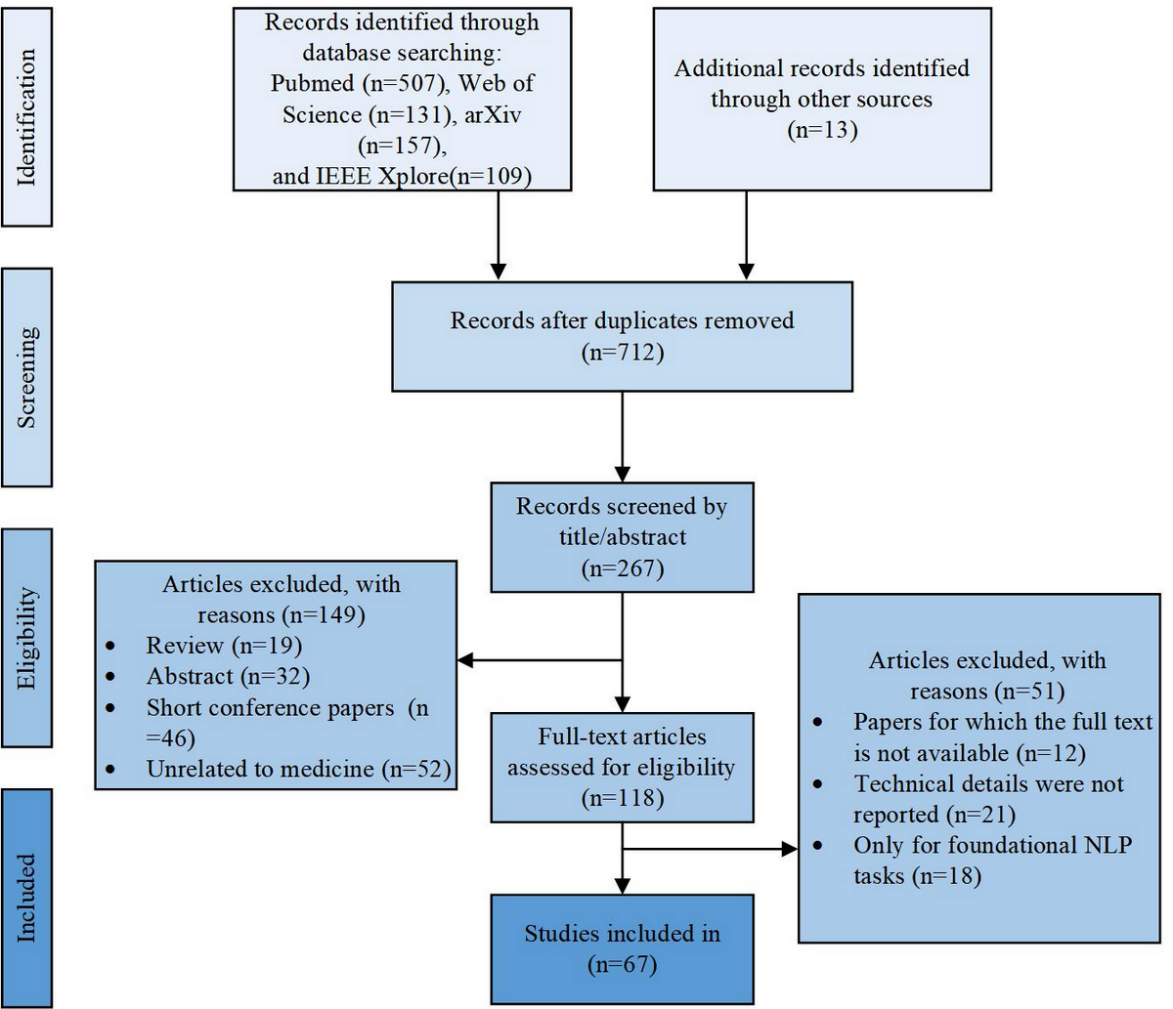
PRISMA flow diagram.

A total of 917 articles were retrieved from the 4 databases, as illustrated in the PRISMA flow diagram (Figure 1). After removing 205 duplicates, 445 articles were excluded based on title and abstract screening. The interrater reliability for the initial screening was high, with a Cohen κ of 0.87, indicating substantial agreement. A total of 118 full-text articles were assessed for eligibility. Ultimately, 67 studies met the inclusion criteria and were selected in this review.

### Quality Appraisal Results of Included Studies

After consensus was reached, the overall adherence rate to MINIMAR across all included studies was 62.3% (Multimedia Appendix 3). The adherence rates for the 4 essential components of MINIMAR, including study population and setting, patient demographics, model architecture, and model evaluation, were 55.25%, 6.56%, 87.30%, and 89.20%, respectively. The high compliance in model architecture and evaluation suggests that the included studies generally reported the technical aspects well. In contrast, the low adherence in patient demographic reporting highlights a limitation in conveying population characteristics, reflecting the proof-of-concept nature of many of the included studies that often did not involve real patient data. Detailed evaluations for each study are provided in Multimedia Appendix 4 [19-85]. We further assessed each study using a self-developed evaluation framework (Multimedia Appendix 5). The average scores were 3.89 (SD 0.42), 2.59 (SD 0.38), and 2.94 (SD 0.18), respectively, with an overall mean score of 9.43 (SD 0.55) out of 10, indicating moderate to high quality but with room for improvement in clinical applicability (Multimedia Appendix 6 [19-85]).

### RAG Technologies Applied to Medical and Nursing Domains

#### Classification of RAG Methodologies

In this review, RAG methodologies were categorized into 5 functional types: knowledge graph (KG)–enhanced RAG, text-based RAG, agentic RAG, multimodal RAG, and plug-and-play RAG frameworks that directly adopt existing tools. Detailed descriptions of each type are provided in Multimedia Appendix 7. A total of 17 studies implemented RAG frameworks enhanced with KGs [19,26,30-46]. Among them, 3 studies used dynamically constructed KGs [26,30,33]. In parallel, 6 studies applied agentic RAG frameworks [20,47-51]. Two studies proposed a multimodal RAG framework that integrates text with other modalities [29,52]. Six studies directly adopted existing RAG plug-and-play frameworks, including LangChain, Pinecone, and NotebookLM, to streamline retrieval and generation [53-58]. The remaining 36 studies fell under the category of text-based RAG. Among them, 2 studies used dynamically evolving knowledge bases rather than static ones [59,60], incorporating real-time sources, such as sensor data or PubMed.

#### KG Construction Approaches

Given the frequent integration of KGs in medical and nursing RAG frameworks, we further examined the methods used for KG construction. Among the 17 studies that adopted KG-RAG frameworks, construction approaches were grouped into 4 major categories. Use of open-source KGs was identified in 7 studies [31,36,37,39,41,42,45]. The most commonly used open-source KGs included the Unified Medical Language System [36,42], along with other structured resources, such as the Scalable Precision Medicine Open Knowledge Engine [31] and the SmartQuerier Oncology KG [37]. A rule-based construction was reported in one study [43]. LLM-assisted methods were used for KG construction in 6 studies [19,30,33,38,40,46]. Deep learning-based approaches were applied in 3 studies [26,34,44].

### RAG Enhancement Strategies Across Pipeline Stages

#### Theoretical Framework for Staging RAG

Herbert A Simon, a pioneer in decision science, proposed a foundational model of the decision-making process that divides it into 3 primary stages [86]. In the intelligence phase, problems are identified and the purpose of the action involving the decision is determined. In the design phase, possible solutions are developed, and alternatives are proposed to address the problem. In the choice phase, the alternative that best meets the decision’s objective is selected. Years later, Turban extended the Simon model by adding a fourth phase called implementation, which focuses on carrying out the chosen solution [87].

**Figure 2:**
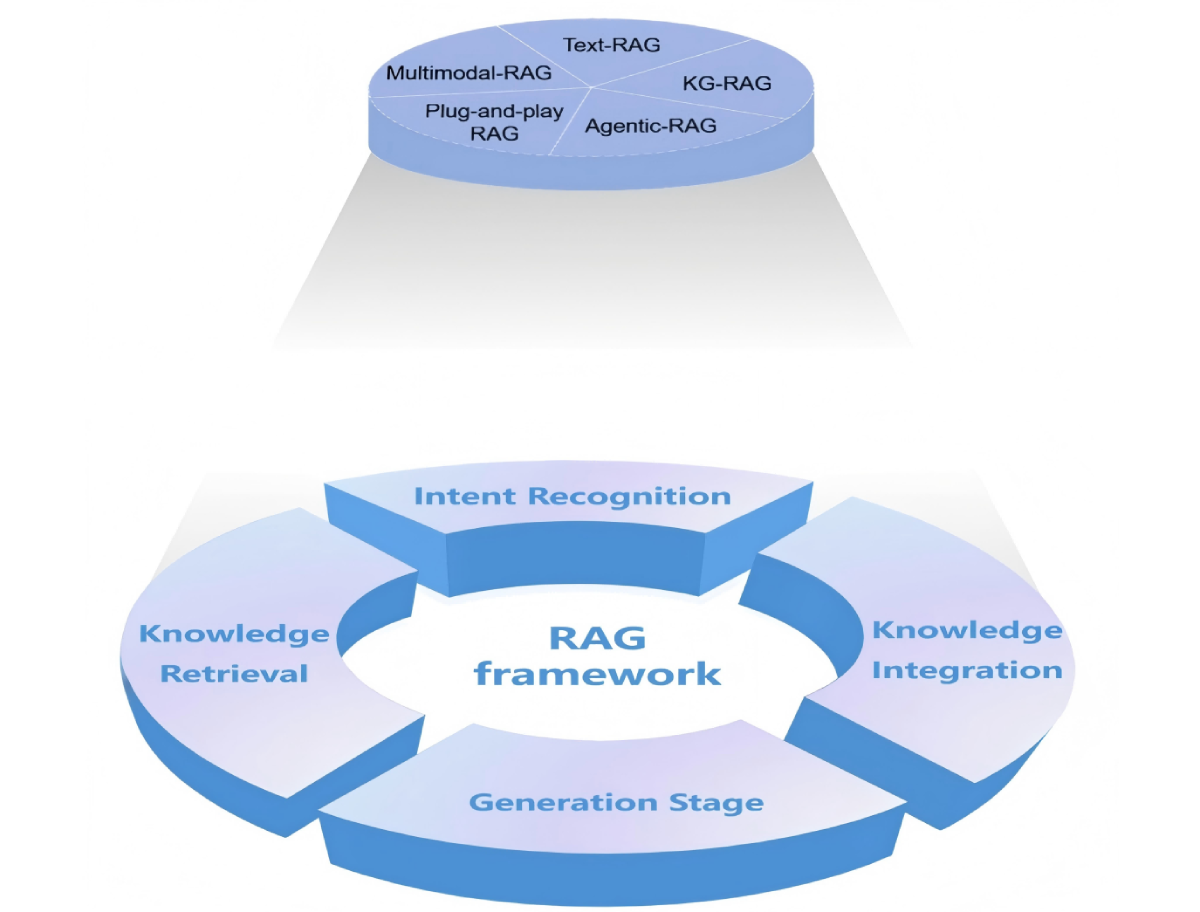
Retrieval-augmented generation classification and stage mapping.

We adopt the Simon decision-making theory to structure the RAG framework, as its staged process aligns with how RAG is applied in clinical and nursing practice. Clinicians and nurses typically identify patient needs, retrieve relevant information, integrate it to form judgments, and generate appropriate interventions. This sequence corresponds to the 4 phases of the decision-making process model: intelligence, design, choice, and implementation. Based on this parallel, we divide the RAG process into 4 stages: intent recognition, knowledge retrieval, knowledge integration, and generation. The 5 categories of RAG and the 4 distinct stages of the RAG system workflow are shown in Figure 2.

#### Enhancements at the Intent Recognition Stage

Among the reviewed studies, approximately half (34/67, 50.7%) applied enhancement techniques at the intent recognition stage. Intent classification, which categorizes user inputs into predefined intent types, was used in 10 studies [29,49-51,61-66]. Query rewriting, which reformulates user queries to improve retrievability or clarity, was implemented in 7 studies [20,43,45,59,67-69]. Query decomposition, which breaks down complex queries into simpler subqueries, was adopted in 4 studies [21,39,40,70]. Medical entity recognition, which extracts clinically relevant terms from user input, appeared in 11 studies [19,22,30,31,33,35-37,41,42,71]. Semantic parsing, which converts natural language into structured meaning representations, was identified in one study [26]. In addition, one study [48] used a hybrid strategy that combined both intent classification and semantic parsing.

#### Enhancements at the Knowledge Retrieval Stage

All the studies were enhanced at the knowledge retrieval stage. In total, 5 distinct retrieval strategies were identified across the included studies. Hybrid retrieval, involving the combination of multiple retrieval mechanisms, was applied in 16 studies [26,29,30,34,39,41,42,44,47,48,50,60,61,68,71,72]. Sparse retrieval, often based on traditional keyword-matching methods or statistical models, such as BM25 or term frequency-inverse document frequency (TF-IDF), was used in 6 studies [33,36,59,62,67,73]. Dense retrieval, which uses neural networks to encode queries and documents into vectors for similarity-based retrieval, was the most frequently adopted individual strategy, appearing in 33 studies [22-25,27,28,31,32,35,38,40,45,46,51,52,63-66,69,70,74-85]. Structured retrieval, which queries schema-based knowledge sources, was adopted in 5 studies [19,20,37,41,43]. Recursive augmented retrieval, which iteratively refines queries based on intermediate outputs, was found in one study [21].

Because of its dominant role among retrieval strategies, dense retrieval was analyzed in greater depth with respect to its implementation components. Specifically, commonly used embedding models included text-embedding-ada-002, text-embedding-3-small, and sentence transformer variants (eg, all-mpnet-base-v2, all-MiniLM-L6-v2), as well as BAAI General Embedding (BGE), GIST-large-embedding-v0, gte-base-zh, and Vertex AI Search. Facebook AI Similarity Search (FAISS) was the most frequently used vector similarity engine, typically using cosine similarity for top-k retrieval. In addition, some studies used custom retrievers specifically designed for biomedical applications, such as MedCPT [21,36,42,63].

#### Enhancements at the Knowledge Integration Stage

At the knowledge integration stage, many studies combined 2 or more methods to enhance accuracy. Among these, reranking was the most commonly applied technique across the included studies. Additional approaches included authenticity verification [20,24,38,43,45,46,51,62,63,65,69,84], semantic consistency control [20,41,44,65,72,80,82], conflict detection [38,41,44,48,49,61], multisource fusion [19,21,24,49,50,52,59,60,62], and structured reasoning [26,30,33,37,40,43,46,47,50,70]. Notably, one study [22] investigated knowledge compression strategies to eliminate redundant content before integration. Multimedia Appendix 8 [19-85] presents the specific techniques adopted at each stage of the RAG pipeline.

#### Enhancements at the Generation Stage

Three primary strategies were identified at the answer-generation stage to enhance the quality and reliability of the model outputs. At this stage, nearly all the reviewed studies used prompt engineering strategies to regulate the output behavior of LLMs, including the structure, tone, and content of the generated responses. Building on this, 19 studies further incorporated chain-of-thought (CoT) prompting, a technique that guides models to perform structured, step-by-step reasoning, thereby enhancing the logical consistency of the generated outputs [21,24,29,30,33,34,39-43,48-50,60,61,63,65,72]. In addition, 3 studies [60,63,76] used self-reflection methods that enabled the model to evaluate and revise its initial responses.

### Reasoning Strategies in RAG Frameworks

Among the reviewed studies, 26 incorporated various reasoning strategies within their RAG frameworks to help the LLMs follow clinical reasoning pathways. Agentic multistage reasoning was adopted in 6 studies [20,47-51]. Five studies [46,61,69,72,79] used CoT prompting, one of which [46] used an iterative refinement variant of CoT. Notably, this form of CoT differs from the one used during the answer-generation stage. In this context, CoT serves as an explicit reasoning framework that structures the model inferential process, rather than merely functioning as a general prompt to elicit step-by-step outputs. Graph-structured reasoning was used in 11 studies [26,30,33,34,37-41,43,70], including 1 study [70] that applied directed acyclic graph-based reasoning.

In addition to the common strategies described above, 4 studies applied more specialized reasoning approaches that closely reflected real-world clinical workflows. Two studies incorporated clinical process–aligned reasoning [24,29], which aims to mimic the step-by-step logic of clinical decision-making. Specifically, MEDPLAN [24] simulated subjective, objective, assessment, and plan-based diagnostic workflows by sequentially generating assessments and treatment plans, while DrHouse [29] adopted an exclusion-based reasoning model that updated disease probabilities through guideline-driven questioning. Two other studies implemented recurrence-based multihop reasoning [21,65], in which reasoning was achieved through iterative query refinement and multistep evidence accumulation. MedRAG [40] simulated multi-round diagnostic reasoning using a proactive questioning mechanism based on differential features, while recurrence generation–augmented retrieval (RGAR) [21] used recursive alignment between conceptual knowledge and patient-specific facts to iteratively refine diagnostic conclusions.

### Application Distribution of RAG in Medical and Nursing Domains

**Figure 3:**
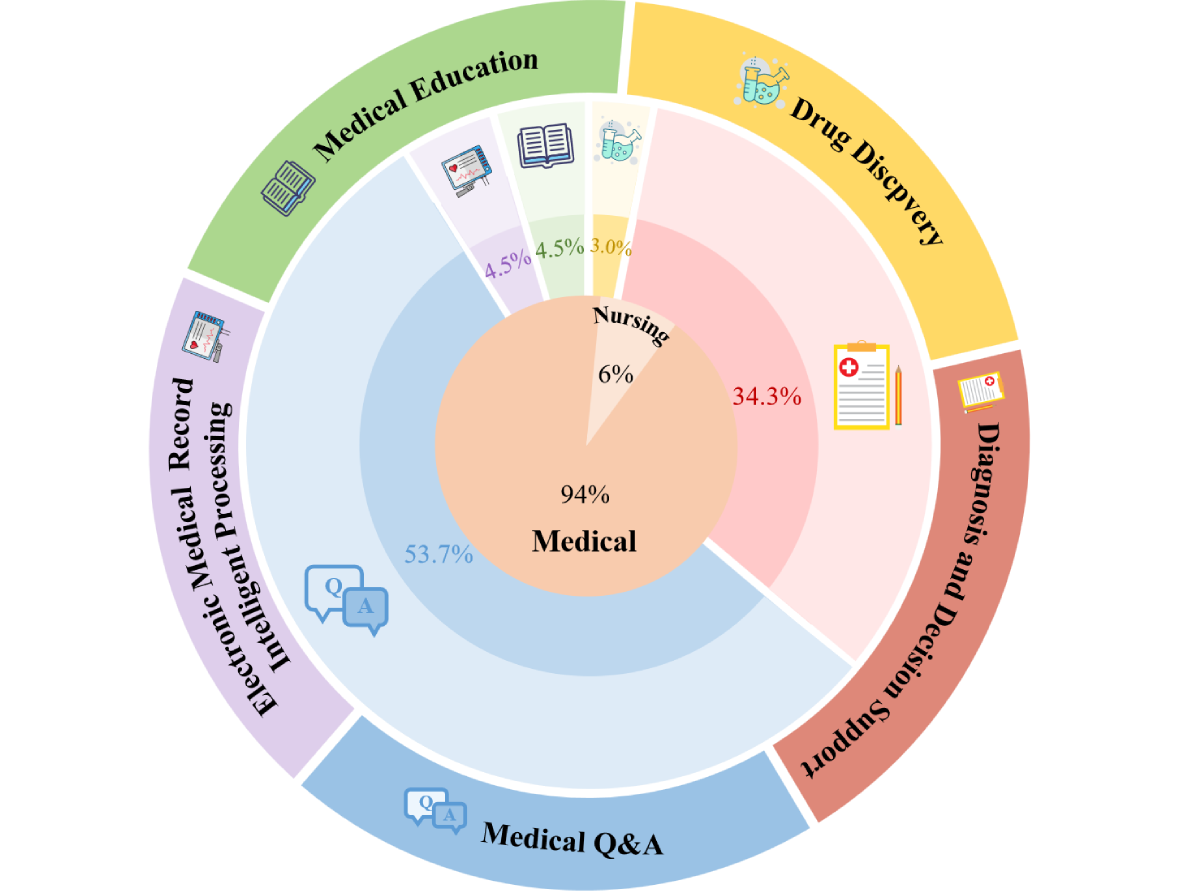
Distribution of application tasks in medical and nursing fields using retrieval-augmented generation.

Of the 67 studies included in this review, 23 (34%) focused on diagnostic and clinical decision support, 36 (54%) addressed medical question answering, 2 (3%) explored drug discovery, 3 (4%) focused on medical education, and 3 (4%) were applied to the intelligent processing of electronic medical records. Crucially, a gap was identified in nursing-focused research. Of the 67 included studies, only 4 (6%) [27,64,76,81] were specifically designed for nursing-related applications, and their scope was limited to question-answering tasks. The distribution of the RAG tasks and their corresponding percentages are shown in Figure 3.

### Sensitivity Analysis

A sensitivity analysis, excluding the 39 preprints, was performed to assess their influence on the conclusions of this review. The reanalysis using only peer-reviewed articles yielded no substantial differences in the distribution of RAG framework types, the proposed workflow stages, or the key findings regarding reasoning support and ethical considerations.

### Ethical Considerations

The medical and nursing domains are among the most highly regulated sectors, governed by principles such as biomedical ethics and stringent data protection regulations. To ensure the responsible deployment of LLMs in health care, ethical concerns must be carefully addressed. These include safeguarding data privacy, enhancing patient safety, and ensuring fairness for patients. Among the 67 studies reviewed, only 9 explicitly addressed patient data privacy. Seven studies [19-25] applied deidentification techniques, one [26] used stratified isolation via triple graph construction by separating patient data into a dedicated layer, and another [27] used an advanced encryption standard with key management services to secure sensitive patient information. Patient safety was a focus in only 1 study [28]; a technique that flags safety concerns was developed, demonstrating zero instances of alarming red flags during testing. Fairness was considered in 2 studies. One study [20] conducted a detailed evaluation of system performance across 32 personality configurations, while another applied [29] a previous probability adjustment to reduce demographic biases.

## Discussion

### Principal Findings

To the best of our knowledge, this review is the first to systematically examine RAG frameworks in the medical and nursing domains, highlighting common practices, current trends, and underexplored areas in the design of domain-specific RAG systems. By categorizing RAG frameworks into text-based, multimodal, agentic, KG-enhanced, and plug-and-play types, we identified key architectural trends. We further divided the RAG framework into 4 stages, namely intent recognition, knowledge retrieval, knowledge integration, and generation, and explored the specific techniques applied at each stage. We observed several notable technological trends: shifting from surface-level matching toward contextualized intent recognition [31,49], from vague semantics toward logic-driven dynamic retrieval [33,37], from passive toward active knowledge retrieval [32,50], and from simple aggregation toward coherent context construction [40,83]. Moreover, although various reasoning strategies have emerged, few systems align with the procedural logic of medical and nursing workflows, highlighting a significant gap between current implementations and domain-specific reasoning needs. Importantly, we also identified a profound imbalance between the medical and nursing applications of RAG, with nursing-specific research remaining sparse and insufficiently explored.

A persistent challenge and research focus is the selection of external knowledge sources, as it directly influences the retrieval accuracy of RAG. This review highlights the widespread adoption of KGs within RAG frameworks, owing to their structured logic capabilities. Furthermore, given the dynamic and evolving nature of patient conditions, research is increasingly focusing on the retrieval of dynamic knowledge sources as a complement to static repositories. For example, one study enabled the integration of real-time sensor data, which proved particularly beneficial for handling complex and evolving patient cases [29]. However, concerns about the quality of external knowledge sources remain a significant challenge. Graph construction is increasingly shifting from deep learning-based methods to LLM-driven generation. Although this approach improves efficiency, it often fails to reflect the specific procedures and accuracy required in medical and nursing workflows [88]. Future work should focus on improving the quality of external knowledge sources. For example, building event-centered cognitive KGs that align with disease progression can support dynamic reasoning and enhance the ability of RAG systems to manage the complexity of real clinical settings.

Most studies focus on the final performance of the system, neglecting the analysis of each development stage. In contrast, this study examined all stages of RAG system development, enabling clinicians and nurses to better understand its internal functioning and take a leading role in guiding its design and implementation. Our study revealed the evolution from keyword-based methods to a deeper semantic understanding in the intent recognition stage. Because of the considerable variability in patient communication styles and health literacy levels, their queries are often diverse and unstandardized, rendering keyword-based methods inadequate for effectively handling patient-generated natural language questions [89]. To address this, recent RAG frameworks have increasingly introduced semantic-focused techniques early in the pipeline, including expansion, disambiguation, and decomposition [90]. However, in real-world clinical practice, these methods often introduce noise and add computational burden, and even small delays can compromise their usefulness in time-critical settings, such as emergency triage and bedside decision-making. Therefore, future work should explore optimization strategies that balance retrieval precision with efficiency, enabling scalable deployment of RAG systems in routine health care settings.

This review identified a notable shift at the knowledge retrieval stage, from ambiguous semantic matching to logic-driven dynamic retrieval. Most current studies still rely heavily on dense retrieval methods based on semantic similarity. Although these methods perform well in capturing general semantic resemblance, they often fail to recognize strict clinical logic, such as negation and hierarchical structures. As a result, they may return outputs that are semantically similar but clinically inconsistent or contextually inappropriate [91,92]. Although recent work has introduced logic-driven dynamic retrieval methods that incorporate clinical reasoning and contextual adaptation into the retrieval process, these approaches still face significant limitations [21]. In particular, current methods often fail to recognize temporal sequences and hierarchical structures that are critical in medical and nursing contexts. Therefore, future research should focus on developing retrieval frameworks capable of deep understanding and using these relationships to provide more accurate and context-aware support.

Our review also identified a trend in the retrieval stage, shifting from passive to active knowledge retrieval. Instead of simply returning relevant content, emerging systems can adjust both what information they retrieve and how they retrieve it, based on real-time contexts, such as changes in the patient’s condition or the history of queries [93]. This proactive retrieval approach holds particular promise for active patient management by providing more timely and context-aware support. Building on this trend, we cautiously speculate that future large models may evolve to proactively interact with the external world and continuously generate feedback without relying entirely on human-provided knowledge [94]. However, such capabilities are still in their early stages. A major challenge that remains is building trust. To gain acceptance in clinical settings, proactive agents must be able to reliably interpret complex situations and clearly explain their actions. Without robust mechanisms for accountability and transparency, they may be perceived as unsafe or untrustworthy. Therefore, the immediate research goal may not be full autonomy, but rather developing “human-in-the-loop” systems in which proactive agents suggest actions or information that clinicians or nurses can quickly validate, modify, or reject, seamlessly integrating AI proactivity with human oversight.

In terms of knowledge integration, most RAG frameworks in our review still follow the approach of directly feeding all retrieved chunks into the language model context [95]. Although simple, this often leads to fragmented, inconsistent, or clinically irrelevant outputs, especially in the high-stakes environments of medicine and nursing [96]. A growing body of research is moving from simple information aggregation to logically coherent context construction. For example, the studies reviewed mention techniques such as evidence reranking, authenticity verification, and knowledge compression, all designed to prioritize high-quality medical knowledge before generation [97]. However, when dealing with multimodal data, these techniques still fail to achieve effective knowledge integration. In real-world clinical scenarios, effective decision-making often requires the synthesis of heterogeneous data types, including text, images, structured records, and real-time sensor signals [98]. Future efforts should focus on frameworks that can effectively align across modalities to support more comprehensive, accurate, and patient-centered outputs.

Reasoning is essential in medical and nursing practice, where professionals must continuously interpret patient condition changes, formulate hypotheses, gather additional information, and identify underlying causes to determine appropriate interventions. LLMs can only truly support clinical work if they acquire this reasoning ability, which is still underdeveloped in current systems. Current research primarily focuses on enhancing the reasoning capabilities of LLMs through prompting techniques. However, these methods are fundamentally constrained by their reliance on associative learning rather than causal inference [99]. While excelling at pattern recognition, they struggle to mimic the abductive or deductive reasoning required in medical diagnosis and nursing care planning. In addition, some studies attempt to model reasoning using annotated clinical formats, such as subjective, objective, assessment, and plan [24]. However, these approaches primarily facilitate implicit pattern imitation rather than explicit learning of causal mechanisms, and struggle to capture the causal relationships embedded in clinical and nursing workflows. To address these limitations, future work should incorporate causal science approaches, such as causal graphs and structural causal models, to constrain model outputs, thereby improving the reasoning performance of LLMs [100].

A central finding of this review is the profound imbalance between the medical and nursing applications of RAG. Although RAG frameworks have been applied across various scenarios, only 6% (4/67) of the included studies focused on the nursing domain, and these were primarily limited to question-answering tasks. Core nursing practices, such as proactive patient management in home care settings, remain largely unexplored [101]. One possible reason is the dominant focus on physician-centered workflows, which has led to a relative lack of resources for nursing applications. Publicly available datasets and evaluation benchmarks, for example, are typically designed around clinician-driven tasks [102]. However, nursing reasoning is as complex as clinical decision-making, involving continuous monitoring, real-time decision-making, and frequent patient interactions [103]. KG-based RAG, which is capable of retrieving 2- or 3-hop entities, is well suited to support such complexity. Furthermore, while medical knowledge systems are relatively well established, nursing still lacks standardized and structured knowledge representations, which hinders the effective integration of nursing knowledge into RAG systems [104]. To truly bridge this gap, we call for a concerted effort that not only advances nursing knowledge modeling and benchmark development but also equips nurses with education on RAG and related AI technologies, thereby enabling more widespread and equitable integration of RAG into nursing practice.

Ethical concerns such as bias, privacy, and safety are critical when applying RAG-based LLMs in the medical and nursing domains [105]. Our review shows that only a small number of studies have attempted to address these issues, highlighting significant room for improvement. Although RAG offers significant potential, its use must be guided by ethical standards to protect patient privacy and ensure safety. For example, connecting to external databases may risk exposing sensitive information such as prescription records [106]. Current mitigation approaches often rely on static safeguards, such as the removal of personally identifiable information and the implementation of role-based access controls [107]. However, the dynamic and context-sensitive nature of clinical privacy often renders existing methods inadequate, highlighting the need for future research to develop more adaptive privacy-preserving mechanisms, such as differential privacy, real-time consent management, and query auditing tools that can respond to evolving regulatory requirements [108]. Beyond privacy, patient safety and algorithmic bias represent major ethical challenges. To ensure safety, RAG-based systems should incorporate proactive measures, such as comprehensive adversarial testing and simulation of edge-case scenarios [109]. At the same time, algorithmic bias, which may exacerbate health disparities, should be mitigated through systematic bias audits, fairness-aware algorithms, and transparent reporting of model performance across diverse demographic groups.

### Limitations

This study has several important limitations. First, it included only English-language literature. Although translating non-English studies could introduce biases or inaccuracies, this exclusion may have led to the omission of relevant research in other languages. Second, preprints were included to capture the most recent developments in this rapidly evolving field. However, as preprints lack peer review, they may overrepresent unvalidated innovations, potentially introducing bias into the findings. Therefore, conclusions drawn from these sources should be considered preliminary, and future reviews may reassess the evidence once these preprints are formally published and peer reviewed. In addition, the number of nursing-focused studies included in the review was relatively small, despite using nursing-specific search terms. Although we conducted supplementary searches of gray literature sources, no additional eligible nursing-related studies were identified. As such, findings related to nursing should be interpreted with caution. Further research is needed to validate and extend these findings within the nursing context. Finally, because of the lack of specialized evaluation tools for the emerging field of RAG, we used MINIMAR for quality assessment. Although not ideal, MINIMAR was the most appropriate available framework for evaluating RAG systems at this stage.

### Conclusions

This review summarizes the current applications and trends of RAG frameworks in the medical and nursing domains. We classified RAG types and analyzed their techniques across 4 functional stages. Although early efforts toward logic-driven reasoning exist, alignment with clinical and nursing workflows remains limited, highlighting a key direction for future research. In addition, we found a profound imbalance between the medical and nursing applications of RAG and call for greater attention to nursing-specific needs.

## Appendix

Multimedia Appendix 1 PRISMA-ScR checklist.

Multimedia Appendix 2 Search strategy.

Multimedia Appendix 3 Overall Minimum Information for Medical AI Reporting (MINIMAR) scores for the included studies.

Multimedia Appendix 4 Detailed item-level Minimum Information for Medical AI Reporting (MINIMAR) scores for each included study.

Multimedia Appendix 5 Self-developed evaluation checklist.

Multimedia Appendix 6 Quality evaluation of the included studies using the self-developed framework.

Multimedia Appendix 7 Descriptions of retrieval-augmented generation stage categories and methods.

Multimedia Appendix 8 Summary of the studies on retrieval-augmented generation methods.

## Abbreviations

AI: artificial intelligence
CoT: chain-of-thought
FAISS: Facebook AI Similarity Search
KG: knowledge graph
LLM: large language model
MINIMAR: Minimum Information for Medical AI Reporting
PRISMA-ScR: Preferred Reporting Items for Systematic Reviews and Meta-Analyses Extension for Scoping Reviews
RAG: retrieval-augmented generation
RGAR: recurrence generation–augmented retrieval
TF-IDF: term frequency-inverse document frequency

## Data Availability

All data generated or analyzed during this study are included in this published paper and its multimedia appendices.
