# Supplementary material for: Improving Large Language Model Applications in the Medical and Nursing Domains With Retrieval-Augmented Generation: Scoping Review
Source: J Med Internet Res. 2025 Oct 21;27:e80557. doi: 10.2196/80557 (PMC12587015; doi:10.2196/80557)
Supplement: Multimedia Appendix 2 [file jmir_v27i1e80557_app2.docx]

**Multimedia Appendix 2. Search strategy**

**Pubmed**

#1 ((large language models[Mesh])) OR (LLMs[Title/Abstract]) OR (transformer models[Title/Abstract])) OR (Generative AI[Title/Abstract])))

#2 (retrieval augmented generation[Mesh])) OR (retrieval[Title/Abstract]) OR (augmented[Title/Abstract])) OR (augmentedgeneration[Title/Abstract]))) OR (RAG[Title/Abstract])

#3 (medicine[Mesh])) OR (healthcare[Title/Abstract]) OR (medical[Title/Abstract])) OR (biomedical[Title/Abstract]))) OR (nurse[Mesh]) OR (nursing[Title/Abstract])) OR (care[Title/Abstract])))

#4 #1 AND #2 AND #3 507

**Web of science**

#1 TS=(large language models OR LLMs OR transformer models OR Generative AI )

#2 TS=(retrieval augmented generation OR retrieval OR augmented OR generation OR RAG)

#3 TS=(medicine OR healthcare OR medical OR biomedical OR nurse OR nursing OR care)

#4 #1 AND #2 AND #3 131

**IEEE Xplore**

“large language models” OR “LLMs” OR “transformer models” OR “Generative AI” AND “retrieval augmented generation” OR “retrieval” OR “augmented” OR “generation” OR “RAG” AND “medicine” OR “healthcare” OR “medical” OR “biomedical” OR “nurse” OR “nursing” OR “care” 109

**arXiv**

“large language models” OR “LLMs” OR “transformer models” OR “Generative AI” AND “retrieval augmented generation” OR “retrieval” OR “augmented” OR “generation” OR “RAG” AND “medicine” OR “healthcare” OR “medical” OR “biomedical” OR “nurse” OR “nursing” OR “care” 157
