# Supplementary material for: Improving Large Language Model Applications in the Medical and Nursing Domains With Retrieval-Augmented Generation: Scoping Review
Source: J Med Internet Res. 2025 Oct 21;27:e80557. doi: 10.2196/80557 (PMC12587015; doi:10.2196/80557)
Supplement: Multimedia Appendix 3 [file jmir_v27i1e80557_app3.docx]

Multimedia Appendix 3 Overall Minimum Information for Medical AI Reporting (MINIMAR) scores for the included studies.

| Total (21 items) | All article (n =67) |
| --- | --- |
| Study population and setting | 55.25% |
| Population | 15(22.4) |
| Setting | 56(83.6) |
| Data source | 67(100) |
| Cohort selection | 10(15.0) |
| Patient demographics | 6.56% |
| Age | 7(10.4) |
| Sex | 7(10.4) |
| Race | 4(6.0) |
| Ethnicity | 3(4.5) |
| Socioeconomic status | 1(1.5) |
| Model architecture | 87.30% |
| Model output | 67(100) |
| Target user | 66(98.5) |
| Data splitting | 65(97.0) |
| Gold standard | 67(100) |
| Model task | 67(100) |
| Model architecture | 67(100) |
| Features | 67(100) |
| Missingness | 2(3.0) |
| Model evaluation | 89.20% |
| Optimization | 67(100) |
| Internal validation | 65(97.0) |
| External validation | 42(62.7) |
| Transparency | 65(97.0) |
| Mean adherence rate | 62.30% |

Note: MINIMAR Minimum Information for Medical AI Reporting
