# Supplementary material for: Improving Large Language Model Applications in the Medical and Nursing Domains With Retrieval-Augmented Generation: Scoping Review
Source: J Med Internet Res. 2025 Oct 21;27:e80557. doi: 10.2196/80557 (PMC12587015; doi:10.2196/80557)
Supplement: Multimedia Appendix 5 [file jmir_v27i1e80557_app5.docx]

Multimedia Appendix 5 Self-developed evaluation checklist.

| Section | Checklist Itsm |
| --- | --- |
| A. Methodological rigor & risk of bias |  |
| A1 | Representativeness of Data Sources: Is the knowledge corpus (e.g., clinical guidelines, EHR data) clearly described and representative of a real-world clinical context? |
| A2 | Validity of Query Set: Are the clinical questions/queries used for evaluation representative and clinically meaningful (e.g., derived from real clinical scenarios rather than simulated)? |
| A3 | Appropriateness of Metrics: Does the study evaluate both retrieval quality (e.g., Recall@K, NDCG) and generation quality (e.g., answer accuracy, hallucination rate, BLEU/ROUGE)? |
| A4 | Baseline Comparison: Is the RAG system compared against a reasonable baseline method (e.g., a vanilla LLM without retrieval, a traditional search system)? |
| B. Clinical relevance & translation potential |  |
| B1 | Clinical Problem Definition: Is the specific clinical problem or need being addressed clearly defined? |
| B2 | Clinical Evaluation: Does the evaluation include an end-to-end assessment by clinical experts (e.g., physicians, nurses) judging utility, accuracy, and safety (not solely automated metrics)? |
| B3 | Error Analysis: Is there an analysis of failure cases, with discussion of the potential clinical implications of such errors? |
| C. Reporting transparency |  |
| C1 | Architecture Description: Is the architecture of the RAG system (including core components: retriever, generator, and their interaction) clearly described? |
| C2 | Data Availability: Are the sources of the knowledge corpus and test sets detailed? Is access provided or explicitly restricted (e.g., for privacy reasons)? |
| C3 | Replicability: Is the code, model, or detailed prompts made available to facilitate replication of the study? |
