# Supplementary material for: Improving Large Language Model Applications in the Medical and Nursing Domains With Retrieval-Augmented Generation: Scoping Review
Source: J Med Internet Res. 2025 Oct 21;27:e80557. doi: 10.2196/80557 (PMC12587015; doi:10.2196/80557)
Supplement: Multimedia Appendix 6 [file jmir_v27i1e80557_app6.docx]

**Multimedia Appendix 6** Quality evaluation of the included studies using the self-developed framework.

| study | A1 | A2 | A3 | A4 | B1 | B2 | B3 | C1 | C2 | C3 |
| --- | --- | --- | --- | --- | --- | --- | --- | --- | --- | --- |
| Zuo et al.[1] | 1 | 0 | 0 | 0 | 1 | 1 | 0 | 1 | 1 | 1 |
| Zhao et al.[2] | 1 | 1 | 1 | 1 | 1 | 1 | 1 | 1 | 1 | 1 |
| Lecu et al.[3] | 1 | 0 | 1 | 1 | 0 | 1 | 1 | 1 | 1 | 1 |
| Zhuang et al.[4] | 1 | 1 | 1 | 1 | 1 | 1 | 0 | 1 | 1 | 1 |
| Matsumoto et al.[5] | 1 | 1 | 1 | 1 | 1 | 1 | 0 | 1 | 1 | 1 |
| Rezaei et al.[6] | 1 | 0 | 1 | 1 | 0 | 1 | 1 | 1 | 1 | 1 |
| Gubanov et al.[7] | 1 | 1 | 1 | 1 | 1 | 1 | 1 | 1 | 1 | 1 |
| Feng et al.[8] | 1 | 1 | 1 | 1 | 1 | 1 | 1 | 1 | 1 | 1 |
| Lu et al.[9] | 1 | 1 | 1 | 1 | 1 | 1 | 0 | 1 | 1 | 1 |
| Yu et al.[10] | 1 | 1 | 1 | 1 | 1 | 1 | 0 | 1 | 1 | 1 |
| Li et al.[11] | 1 | 1 | 1 | 1 | 1 | 1 | 0 | 1 | 1 | 1 |
| Li et al.[12] | 1 | 1 | 1 | 1 | 1 | 1 | 0 | 1 | 1 | 1 |
| Long et al.[13] | 1 | 1 | 1 | 1 | 1 | 1 | 1 | 1 | 1 | 1 |
| Hou et al.[14] | 1 | 1 | 1 | 1 | 1 | 1 | 1 | 1 | 1 | 1 |
| Zheng et al.[15] | 1 | 1 | 1 | 1 | 1 | 1 | 1 | 1 | 1 | 1 |
| Hsu et al.[16] | 1 | 1 | 1 | 1 | 1 | 0 | 1 | 1 | 1 | 1 |
| Dou et al.[17] | 1 | 1 | 1 | 1 | 1 | 1 | 1 | 1 | 1 | 1 |
| Low et al.[18] | 1 | 1 | 1 | 1 | 1 | 1 | 0 | 1 | 1 | 1 |
| Kaczmarek et al.[19] | 1 | 1 | 1 | 1 | 1 | 1 | 1 | 1 | 1 | 1 |
| Feng et al.[20] | 1 | 1 | 1 | 1 | 1 | 1 | 1 | 1 | 1 | 1 |
| Lee et al.[21] | 1 | 1 | 1 | 1 | 1 | 1 | 1 | 1 | 1 | 1 |
| Chu et al.[22] | 1 | 1 | 1 | 1 | 1 | 1 | 1 | 1 | 1 | 1 |
| Jia et al.[23] | 1 | 1 | 1 | 1 | 1 | 1 | 1 | 1 | 1 | 1 |
| Liang et al.[24] | 1 | 1 | 1 | 1 | 1 | 1 | 1 | 1 | 1 | 1 |
| Shi et al.[25] | 1 | 1 | 1 | 1 | 1 | 1 | 1 | 1 | 1 | 1 |
| Kim et al.[26] | 1 | 1 | 1 | 1 | 1 | 1 | 1 | 1 | 1 | 1 |
| Agrawal et al.[27] | 1 | 1 | 1 | 1 | 1 | 1 | 0 | 1 | 1 | 1 |
| Li et al.[28] | 1 | 1 | 1 | 1 | 1 | 1 | 0 | 1 | 1 | 1 |
| Gao et al.[29] | 1 | 1 | 1 | 1 | 1 | 1 | 1 | 1 | 1 | 1 |
| Chen et al.[30] | 1 | 1 | 1 | 1 | 1 | 1 | 1 | 1 | 1 | 1 |
| Wu et al.[31] | 1 | 1 | 1 | 1 | 1 | 0 | 0 | 1 | 1 | 1 |
| Silva et al.[32] | 1 | 1 | 1 | 1 | 1 | 1 | 1 | 1 | 1 | 0 |
| Dietrich et al.[33] | 1 | 1 | 1 | 1 | 1 | 1 | 1 | 1 | 1 | 0 |
| Chen et al.[34] | 1 | 1 | 1 | 1 | 1 | 1 | 1 | 1 | 1 | 1 |
| Cho et al.[35] | 1 | 1 | 1 | 1 | 1 | 1 | 1 | 1 | 1 | 1 |
| Soman et al.[36] | 1 | 1 | 1 | 1 | 1 | 1 | 1 | 1 | 1 | 1 |
| Yang et al.[37] | 1 | 1 | 1 | 1 | 1 | 1 | 1 | 1 | 1 | 1 |
| Wu et al.[38] | 1 | 1 | 1 | 1 | 1 | 1 | 1 | 1 | 1 | 1 |
| Matsumoto et al.[39] | 1 | 1 | 1 | 1 | 1 | 1 | 0 | 1 | 1 | 1 |
| Yang et al.[40] | 1 | 1 | 1 | 1 | 1 | 0 | 0 | 1 | 1 | 1 |
| Rau et al.[41] | 1 | 1 | 1 | 1 | 1 | 1 | 0 | 1 | 1 | 1 |
| Aguzzi et al.[42] | 1 | 1 | 1 | 1 | 1 | 1 | 1 | 1 | 1 | 1 |
| Luo et al.[43] | 1 | 1 | 1 | 1 | 1 | 1 | 1 | 1 | 1 | 1 |
| Jeong et al.[44] | 1 | 1 | 1 | 1 | 1 | 1 | 1 | 1 | 1 | 1 |
| Li et al.[45] | 1 | 1 | 1 | 1 | 1 | 1 | 1 | 1 | 1 | 1 |
| Chen et al.[46] | 1 | 1 | 1 | 1 | 1 | 1 | 1 | 1 | 1 | 1 |
| Fu et al.[47] | 1 | 1 | 1 | 1 | 1 | 1 | 1 | 1 | 1 | 1 |
| Klang et al.[48] | 1 | 1 | 1 | 1 | 1 | 1 | 1 | 1 | 1 | 1 |
| Xu et al.[49] | 1 | 1 | 1 | 1 | 1 | 1 | 1 | 1 | 1 | 1 |
| Yazaki et al.[50] | 1 | 1 | 1 | 1 | 1 | 1 | 1 | 1 | 1 | 1 |
| Zhou et al.[51] | 1 | 1 | 0 | 1 | 1 | 1 | 1 | 1 | 1 | 1 |
| Lammer et al.[52] | 1 | 1 | 1 | 1 | 1 | 1 | 0 | 1 | 1 | 1 |
| Steybe et al.[53] | 1 | 1 | 1 | 1 | 1 | 1 | 0 | 1 | 1 | 1 |
| Sohn et al.[54] | 1 | 1 | 1 | 1 | 1 | 1 | 0 | 1 | 1 | 1 |
| Long et al.[55] | 1 | 1 | 1 | 1 | 1 | 1 | 0 | 1 | 1 | 1 |
| Shi et al.[56] | 1 | 1 | 1 | 1 | 1 | 1 | 0 | 1 | 1 | 1 |
| Tsai et al.[57] | 1 | 1 | 1 | 1 | 1 | 1 | 1 | 1 | 1 | 1 |
| Liu et al.[58] | 1 | 1 | 1 | 1 | 1 | 1 | 1 | 1 | 1 | 1 |
| Zhan et al.[59] | 1 | 1 | 1 | 1 | 1 | 1 | 1 | 1 | 1 | 1 |
| Hammane et al.[60] | 1 | 1 | 1 | 1 | 1 | 1 | 0 | 1 | 1 | 1 |
| Xiong et al.[61] | 1 | 1 | 1 | 1 | 1 | 1 | 1 | 1 | 1 | 1 |
| Bing€ol et al.[62] | 1 | 1 | 1 | 1 | 1 | 1 | 1 | 1 | 1 | 1 |
| Johno et al.[63] | 1 | 1 | 0 | 1 | 1 | 0 | 1 | 1 | 0 | 0 |
| Owoyemi et al.[64] | 1 | 1 | 1 | 1 | 1 | 1 | 1 | 1 | 1 | 1 |
| Sun et al.[65] | 1 | 1 | 1 | 1 | 1 | 1 | 0 | 1 | 1 | 1 |
| Tozuka et al.[66] | 1 | 1 | 1 | 1 | 1 | 1 | 1 | 1 | 1 | 1 |
| Ge et al.[67] | 1 | 1 | 1 | 1 | 1 | 1 | 1 | 1 | 1 | 1 |

Note: A1:Representativeness of Data Sources; A2:Validity of Query Set; A3: Appropriateness of Metrics; A4: Baseline Comparison; B1:Clinical Problem Definition; B2: Clinical Evaluation; B3: Error Analysis; C1:Architecture Description; C2: Data Availability;C3: Replicability.

**References**

1. Zuo K, Jiang Y, Mo F, Lio PJae-p. KG4Diagnosis: A Hierarchical Multi-Agent LLM Framework with Knowledge Graph Enhancement for Medical Diagnosis2024 December 01, 2024:[arXiv:2412.16833 p.]. Available from: <https://ui.adsabs.harvard.edu/abs/2024arXiv241216833Z>.

2. Zhao X, Liu S, Yang S-Y, Miao CJae-p. MedRAG: Enhancing Retrieval-augmented Generation with Knowledge Graph-Elicited Reasoning for Healthcare Copilot2025 February 01, 2025:[arXiv:2502.04413 p.]. Available from: <https://ui.adsabs.harvard.edu/abs/2025arXiv250204413Z>.

3. Lecu A, Groza A, Hawizy LJae-p. Knowledge Graph-Driven Retrieval-Augmented Generation: Integrating Deepseek-R1 with Weaviate for Advanced Chatbot Applications2025 February 01, 2025:[arXiv:2502.11108 p.]. Available from: <https://ui.adsabs.harvard.edu/abs/2025arXiv250211108L>.

4. Zhuang Y, Yu L, Jiang N, Ge Y. TCM-KLLaMA: Intelligent generation model for Traditional Chinese Medicine Prescriptions based on knowledge graph and large language model. Computers in biology and medicine. 2025;189:109887. Epub 2025/03/09. doi: 10.1016/j.compbiomed.2025.109887. PubMed PMID: 40056842.

5. Matsumoto N, Choi H, Moran J, Hernandez ME, Venkatesan M, Li X, et al. ESCARGOT: an AI agent leveraging large language models, dynamic graph of thoughts, and biomedical knowledge graphs for enhanced reasoning. Bioinformatics (Oxford, England). 2025;41(2). Epub 2025/01/23. doi: 10.1093/bioinformatics/btaf031. PubMed PMID: 39842860; PubMed Central PMCID: PMCPMC11796095.

6. Rezaei MR, Saadati Fard R, Parker J, Krishnan RG, Lankarany MJae-p. Adaptive Knowledge Graphs Enhance Medical Question Answering: Bridging the Gap Between LLMs and Evolving Medical Knowledge2025 February 01, 2025:[arXiv:2502.13010 p.]. Available from: <https://ui.adsabs.harvard.edu/abs/2025arXiv250213010R>.

7. Gubanov M, Pyayt A, Karolak AJae-p. CancerKG.ORG A Web-scale, Interactive, Verifiable Knowledge Graph-LLM Hybrid for Assisting with Optimal Cancer Treatment and Care2024 December 01, 2024:[arXiv:2501.00223 p.]. Available from: <https://ui.adsabs.harvard.edu/abs/2025arXiv250100223G>.

8. Feng Y, Zhou L, Ma C, Zheng Y, He R, Li Y. Knowledge graph-based thought: a knowledge graph-enhanced LLM framework for pan-cancer question answering. GigaScience. 2025;14. Epub 2025/01/08. doi: 10.1093/gigascience/giae082. PubMed PMID: 39775838; PubMed Central PMCID: PMCPMC11702363.

9. Lu K, Liang Z, Pan D, Zhang S, Wu X, Chen W, et al. Med-R $^ 2$: Crafting Trustworthy LLM Physicians through Retrieval and Reasoning of Evidence-Based Medicine. 2025.

10. Yu H, Zhou J, Li L, Chen S, Gallifant J, Shi A, et al. AIPatient: Simulating Patients with EHRs and LLM Powered Agentic Workflow. 2024.

11. Li F, Chen Y, Liu H, Yang R, Yuan H, Jiang Y, et al. MKG-Rank: Enhancing Large Language Models with Knowledge Graph for Multilingual Medical Question Answering. 2025.

12. Li M, Kilicoglu H, Xu H, Zhang RJapa. BiomedRAG: A retrieval augmented large language model for biomedicine. arXiv. 2024.

13. Long C, Subburam D, Lowe K, Santos A, Zhang J, Hwang S, et al. ChatENT: Augmented Large Language Model for Expert Knowledge Retrieval in Otolaryngology-Head and Neck Surgery. medRxiv 2023. 2023.

14. Hou Y, Bishop JR, Liu H, Zhang RJJoMIR. Improving Dietary Supplement Information Retrieval: Development of a Retrieval-Augmented Generation System With Large Language Models. 2025;27:e67677.

15. Zheng Y, Yan Y, Chen S, Cai Y, Ren K, Liu Y, et al. Integrating retrieval-augmented generation for enhanced personalized physician recommendations in web-based medical services: model development study. 2025;13:1501408.

16. Hsu H-L, Dao C-T, Wang L, Shuai Z, Phan TNM, Ding J-E, et al. MedPlan: A Two-Stage RAG-Based System for Personalized Medical Plan Generation. 2025.

17. Dou C, Zhang Y, Jin Z, Jiao W, Zhao H, Zhao Y, et al. Enhancing LLM Generation with Knowledge Hypergraph for Evidence-Based Medicine2025 March 01, 2025:[arXiv:2503.16530 p.]. Available from: <https://ui.adsabs.harvard.edu/abs/2025arXiv250316530D>.

18. Low CH, Wang Z, Zhang T, Zeng Z, Zhuo Z, Mazomenos EB, et al. SurgRAW: Multi-Agent Workflow with Chain-of-Thought Reasoning for Surgical Intelligence2025 March 01, 2025:[arXiv:2503.10265 p.]. Available from: <https://ui.adsabs.harvard.edu/abs/2025arXiv250310265L>.

19. Kaczmarek JI, Pokrywka J, Biedalak K, Kurzyp G, Grzybowski ŁJae-p. Optimizing Retrieval-Augmented Generation of Medical Content for Spaced Repetition Learning2025 February 01, 2025:[arXiv:2503.01859 p.]. Available from: <https://ui.adsabs.harvard.edu/abs/2025arXiv250301859K>.

20. Feng H, Yin Y, Reynares E, Nanavati JJapa. OntologyRAG: Better and Faster Biomedical Code Mapping with Retrieval-Augmented Generation (RAG) Leveraging Ontology Knowledge Graphs and Large Language Models. 2025.

21. Lee N, De Brouwer E, Hajiramezanali E, Park C, Scalia GJapa. RAG-Enhanced Collaborative LLM Agents for Drug Discovery. 2025.

22. Chu Y-W, Zhang K, Malon C, Renqiang Min MJae-p. Reducing Hallucinations of Medical Multimodal Large Language Models with Visual Retrieval-Augmented Generation2025 February 01, 2025:[arXiv:2502.15040 p.]. Available from: <https://ui.adsabs.harvard.edu/abs/2025arXiv250215040C>.

23. Jia M, Duan J, Song Y, Wang JJae-p. FIND: Fine-grained Information Density Guided Adaptive Retrieval-Augmented Generation for Disease Diagnosis2025 February 01, 2025:[arXiv:2502.14614 p.]. Available from: <https://ui.adsabs.harvard.edu/abs/2025arXiv250214614J>.

24. Liang S, Zhang L, Zhu H, Wang W, He Y, Zhou DJae-p. RGAR: Recurrence Generation-augmented Retrieval for Factual-aware Medical Question Answering2025 February 01, 2025:[arXiv:2502.13361 p.]. Available from: <https://ui.adsabs.harvard.edu/abs/2025arXiv250213361L>.

25. Shi Y, Yang T, Chen C, Li Q, Liu T, Li X, et al. SearchRAG: Can Search Engines Be Helpful for LLM-based Medical Question Answering? 2025.

26. Kim SJae-p. MedBioLM: Optimizing Medical and Biological QA with Fine-Tuned Large Language Models and Retrieval-Augmented Generation2025 February 01, 2025:[arXiv:2502.03004 p.]. Available from: <https://ui.adsabs.harvard.edu/abs/2025arXiv250203004K>.

27. Mangeshkumar Agrawal A, Pandurang Shinde R, Bhukya VK, Chakraborty A, Bharat Shah S, Shukla T, et al. Conversation AI Dialog for Medicare powered by Finetuning and Retrieval Augmented Generation2025 February 01, 2025:[arXiv:2502.02249 p.]. Available from: <https://ui.adsabs.harvard.edu/abs/2025arXiv250202249M>.

28. Li Z, 'Anthony' Chen X, Jeon YJae-p. GraPPI: A Retrieve-Divide-Solve GraphRAG Framework for Large-scale Protein-protein Interaction Exploration2025 January 01, 2025:[arXiv:2501.16382 p.]. Available from: <https://ui.adsabs.harvard.edu/abs/2025arXiv250116382L>.

29. Gao F, Zhao X, Xia D, Zhou Z, Yang R, Lu J, et al. HealthGenie: Empowering Users with Healthy Dietary Guidance through Knowledge Graph and Large Language Models. 2025.

30. Chen Y, Sun P, Li X, Chu XJapa. MRD-RAG: Enhancing Medical Diagnosis with Multi-Round Retrieval-Augmented Generation. 2025.

31. Wu J, Deng W, Li X, Liu S, Mi T, Peng Y, et al. MedReason: Eliciting Factual Medical Reasoning Steps in LLMs via Knowledge Graphs. 2025.

32. Silva R, Gomes LJCiB, Medicine. An adaptive language model-based intelligent medication assistant for the decision support of antidepressant prescriptions. 2025;190:110065.

33. Dietrich N, Stubbert BJCAoRJ. Evaluating Adherence to Canadian Radiology Guidelines for Incidental Hepatobiliary Findings Using RAG-Enabled LLMs. 2025:08465371251323124.

34. Chen Z, Liao Y, Jiang S, Wang P, Guo Y, Wang Y, et al. Towards Omni-RAG: Comprehensive Retrieval-Augmented Generation for Large Language Models in Medical Applications. 2025.

35. Cho J, Lee GGJapa. K-COMP: Retrieval-Augmented Medical Domain Question Answering With Knowledge-Injected Compressor. 2025.

36. Soman K, Rose PW, Morris JH, Akbas RE, Smith B, Peetoom B, et al. Biomedical knowledge graph-optimized prompt generation for large language models. 2024;40(9):btae560.

37. Yang R, Liu H, Marrese-Taylor E, Zeng Q, Ke YH, Li W, et al. KG-Rank: Enhancing Large Language Models for Medical QA with Knowledge Graphs and Ranking Techniques2024 March 01, 2024:[arXiv:2403.05881 p.]. Available from: <https://ui.adsabs.harvard.edu/abs/2024arXiv240305881Y>.

38. Wu J, Zhu J, Qi Y, Chen J, Xu M, Menolascina F, et al. Medical Graph RAG: Towards Safe Medical Large Language Model via Graph Retrieval-Augmented Generation2024 August 01, 2024:[arXiv:2408.04187 p.]. Available from: <https://ui.adsabs.harvard.edu/abs/2024arXiv240804187W>.

39. Matsumoto N, Moran J, Choi H, Hernandez ME, Venkatesan M, Wang P, et al. KRAGEN: a knowledge graph-enhanced RAG framework for biomedical problem solving using large language models. Bioinformatics (Oxford, England). 2024;40(6). Epub 2024/06/03. doi: 10.1093/bioinformatics/btae353. PubMed PMID: 38830083; PubMed Central PMCID: PMCPMC11164829.

40. Yang B, Jiang S, Xu L, Liu K, Li H, Xing G, et al. DrHouse: An LLM-empowered Diagnostic Reasoning System through Harnessing Outcomes from Sensor Data and Expert Knowledge2024 May 01, 2024:[arXiv:2405.12541 p.]. Available from: <https://ui.adsabs.harvard.edu/abs/2024arXiv240512541Y>.

41. Rau S, Rau A, Nattenmüller J, Fink A, Bamberg F, Reisert M, et al. A retrieval-augmented chatbot based on GPT-4 provides appropriate differential diagnosis in gastrointestinal radiology: a proof of concept study. European radiology experimental. 2024;8(1):60. Epub 2024/05/17. doi: 10.1186/s41747-024-00457-x. PubMed PMID: 38755410; PubMed Central PMCID: PMCPMC11098977.

42. Aguzzi G, Magnini M, Salcuni GP, Ferretti S, Montagna S, editors. Applying Retrieval-Augmented Generation on Open LLMs for a Medical Chatbot Supporting Hypertensive Patients. Proceedings of the 3rd AIxIA Workshop on Artificial Intelligence ForHealthcare (HC@ AIxIA 2024) co-located with the 23rd InternationalConference of the Italian Association for Artificial Intelligence (AIxIA 2024), Bolzano, Italy, 27-28 November 2024; 2024: CEUR-WS. org.

43. Luo MJ, Pang J, Bi S, Lai Y, Zhao J, Shang Y, et al. Development and Evaluation of a Retrieval-Augmented Large Language Model Framework for Ophthalmology. JAMA ophthalmology. 2024;142(9):798-805. Epub 2024/07/18. doi: 10.1001/jamaophthalmol.2024.2513. PubMed PMID: 39023885; PubMed Central PMCID: PMCPMC11258636.

44. Jeong M, Sohn J, Sung M, Kang JJB. Improving medical reasoning through retrieval and self-reflection with retrieval-augmented large language models. 2024;40(Supplement_1):i119-i29.

45. Li Y, Zhao J, Li M, Dang Y, Yu E, Li J, et al. RefAI: a GPT-powered retrieval-augmented generative tool for biomedical literature recommendation and summarization. 2024;31(9):2030-9.

46. Chen X, Zhao Z, Zhang W, Xu P, Wu Y, Xu M, et al. EyeGPT for Patient Inquiries and Medical Education: Development and Validation of an Ophthalmology Large Language Model. 2024;26:e60063.

47. Fu Z, Fu S, Huang Y, He W, Zhong Z, Guo Y, et al. Application of large language model combined with retrieval enhanced generation technology in digestive endoscopic nursing. 2024;11:1500258.

48. Klang E, Tessler I, Apakama DU, Abbott E, Glicksberg BS, Arnold M, et al. Assessing Retrieval-Augmented Large Language Model Performance in Emergency Department ICD-10-CM Coding Compared to Human Coders. 2024.

49. Xu R, Hong Y, Zhang F, Xu HJSR. Evaluation of the integration of retrieval-augmented generation in large language model for breast cancer nursing care responses. 2024;14(1):30794.

50. Yazaki M, Maki S, Furuya T, Inoue K, Nagai K, Nagashima Y, et al. Emergency patient triage improvement through a retrieval-augmented generation enhanced large-scale language model. 2024:1-7.

51. Zhou Q, Liu C, Duan Y, Sun K, Li Y, Kan H, et al. GastroBot: a Chinese gastrointestinal disease chatbot based on the retrieval-augmented generation. 2024;11:1392555.

52. Lammert J, Dreyer T, Mathes S, Kuligin L, Borm KJ, Schatz UA, et al. Expert-guided large language models for clinical decision support in precision oncology. 2024;8:e2400478.

53. Steybe D, Poxleitner P, Aljohani S, Herlofson BB, Nicolatou-Galitis O, Patel V, et al. Evaluation of a context-aware chatbot using retrieval-augmented generation for answering clinical questions on medication-related osteonecrosis of the jaw. 2025.

54. Sohn J, Park Y, Yoon C, Park S, Hwang H, Sung M, et al. Rationale-Guided Retrieval Augmented Generation for Medical Question Answering. 2024.

55. Long C, Liu Y, Ouyang C, Yu YJapa. Bailicai: A Domain-Optimized Retrieval-Augmented Generation Framework for Medical Applications. 2024.

56. Shi Y, Xu S, Yang T, Liu Z, Liu T, Li Q, et al. MKRAG: Medical Knowledge Retrieval Augmented Generation for Medical Question Answering. 2023.

57. Tsai H-C, Chen M-W, Wang J-F, editors. AI-Enhanced Virtual Nursing Systems: Revolutionizing Patient Education in Modern Healthcare. 2024 International Conference on Orange Technology (ICOT); 2024: IEEE.

58. Liu Y-K, Tsai Y-C, editors. Explainable AI for Trustworthy Clinical Decision Support: A Case-Based Reasoning System for Nursing Assistants. 2024 IEEE International Conference on Big Data (BigData); 2024: IEEE.

59. Zhan B, Li A, Yang X, He D, Duan Y, Yan S, editors. RARoK: Retrieval-Augmented Reasoning on Knowledge for Medical Question Answering. 2024 IEEE International Conference on Bioinformatics and Biomedicine (BIBM); 2024: IEEE.

60. Hammane Z, Ben-Bouazza F-E, Fennan A, editors. SelfRewardRAG: enhancing medical reasoning with retrieval-augmented generation and self-evaluation in large language models. 2024 International Conference on Intelligent Systems and Computer Vision (ISCV); 2024: IEEE.

61. Xiong L, Zeng Q, Deng W, Luo W, Liu R. A novel approach to nursing clinical intelligent decision-making: integration of large language models and local knowledge bases. 2023.

62. Bingöl FG, Ağagündüz D, Bingol MCJJoRN. Accuracy of Current Large Language Models and the Retrieval-Augmented Generation Model in Determining Dietary Principles in Chronic Kidney Disease. 2025.

63. Johno H, Johno Y, Amakawa A, Sato J, Tozuka R, Komaba A, et al. Enhancing Pancreatic Cancer Staging with Large Language Models: The Role of Retrieval-Augmented Generation2025 March 01, 2025:[arXiv:2503.15664 p.]. Available from: <https://ui.adsabs.harvard.edu/abs/2025arXiv250315664J>.

64. AI A, Owoyemi J, Abubakar S, Owoyemi A, Togunwa TO, Madubuko FC, et al. Open-Source Retrieval Augmented Generation Framework for Retrieving Accurate Medication Insights from Formularies for African Healthcare Workers2025 January 01, 2025:[arXiv:2502.15722 p.]. Available from: <https://ui.adsabs.harvard.edu/abs/2025arXiv250215722A>.

65. Sun Q, Xie J, Ye N, Gu Q, Guo SJae-p. Enhancing Nursing and Elderly Care with Large Language Models: An AI-Driven Framework2024 December 01, 2024:[arXiv:2412.09946 p.]. Available from: <https://ui.adsabs.harvard.edu/abs/2024arXiv241209946S>.

66. Tozuka R, Johno H, Amakawa A, Sato J, Muto M, Seki S, et al. Application of NotebookLM, a large language model with retrieval-augmented generation, for lung cancer staging. 2024:1-7.

67. Ge J, Sun S, Owens J, Galvez V, Gologorskaya O, Lai JC, et al. Development of a liver disease-specific large language model chat interface using retrieval-augmented generation. Hepatology (Baltimore, Md). 2024;80(5):1158-68. Epub 2024/03/07. doi: 10.1097/hep.0000000000000834. PubMed PMID: 38451962; PubMed Central PMCID: PMCPMC11706764.
