# Supplementary material for: Improving Large Language Model Applications in the Medical and Nursing Domains With Retrieval-Augmented Generation: Scoping Review
Source: J Med Internet Res. 2025 Oct 21;27:e80557. doi: 10.2196/80557 (PMC12587015; doi:10.2196/80557)
Supplement: Multimedia Appendix 7 [file jmir_v27i1e80557_app7.docx]

| Multimedia Appendix 7. Descriptions of retrieval-augmented generation stage categories and methods. | |
| --- | --- |
| Concept | Brief Description |
| **RAG^a^** | RAG refers to retrieval from external knowledge sources, generation with both internal and external knowledge, and the critical process of knowledge integration that bridges these elements. |
| **Intent recognition** | Intent Recognition refers to identifying the user’s underlying query intent in order to guide the subsequent retrieval and generation processes toward relevant and targeted information. |
| **Knowledge retrieval** | Knowledge Retrieval refers to the step of retrieving relevant external knowledge from structured or unstructured sources based on the recognized intent. |
| **Knowledge integration** | Knowledge Integration refers to integrate the retrieved external knowledge with the internal knowledge of the generation model to enhance the output accuracy and coherence. |
| **Generation stage** | Generation Stage refers to incorporating both internal and external knowledge to convert inputs into coherent and pertinent outputs. |
| **Text-based RAG** | Text-based RAG refers to a retrieval-augmented generation framework that retrieves unstructured textual documents (e.g., web passages, articles) to support the generation process with relevant content. |
| **Knowledge graph-enhanced RAG** | Knowledge Graph-enhanced RAG refers to incorporating structured information from knowledge graphs into the retrieval and generation pipeline, enabling more precise and semantically rich responses. |
| **Agentic RAG** | Agentic RAG refers to the integration of autonomous agents with RAG technology, enabling capabilities such as planning, tool use, and multi-step reasoning to actively retrieve and apply knowledge in complex tasks. |
| **Multimodal RAG** | Multimodal RAG refers to extending traditional text-based RAG systems by incorporating multiple modalities including image, audio, video, etc. |
| **Plug-and-play RAG** | Plug-and-play RAG refers to directly using existing RAG frameworks or platforms—such as LangChain, Pinecone, or NotebookLM—to rapidly build and deploy retrieval-augmented applications without redesigning core components. |
| **Intent Classification** | Determines the main intent category of a user query. |
| **Query Decomposition** | Breaks down complex queries into smaller, more manageable sub-questions. |
| **Medical Entity Recognition** | Identifies and extracts medical entities, such as diseases, drugs, or diagnostic tests, from the query. |
| **Query Reformulation** | Rewrites the query to better match retrieval system requirements. |
| **Semantic Parsing** | Converts natural language queries into structured, machine-interpretable semantic representations. |
| **Structured Search** | Retrieves information from structured data sources such as databases or knowledge graphs. |
| **Dense Search** | Retrieves information based on semantic similarity using vector representations. |
| **Sparse Search** | Retrieves information using keyword-based sparse representations. |
| **Hybrid Search** | Combines sparse and dense retrieval to improve recall. |
| **Recursive Augmented Retrieval** | Iteratively refines retrieval using results from previous searches. |
| **Conflict Detection** | Detects contradictions or conflicts between knowledge from different sources. |
| **Multi-source Fusion** | Merges knowledge from multiple sources. |
| **Structured Reasoning** | Performs reasoning based on structured knowledge such as knowledge graphs. |
| **Re-ranking** | Reorders retrieved results to optimize relevance. |
| **Authenticity Verification** | Verifies the authenticity and reliability of the retrieved knowledge. |
| **Semantic Consistency Control** | Ensures generated content is semantically consistent with input knowledge. |
| **Knowledge Compression** | Compresses knowledge to retain key information while reducing redundancy. |
| **Chain-of-Thought** | Generates answers through explicit multi-step reasoning chains. |
| **Self-reflection** | Self-checks and revises potential errors during generation. |
| **Output Control** | Controls the format, style, or scope of the generated output using prompt. |

^a^RAG: Retrieval-Augmented Generation
